# Co-infection of Influenza B and Streptococci causing severe pneumonia and septic shock in healthy women

**DOI:** 10.1186/1471-2334-10-308

**Published:** 2010-10-27

**Authors:** Timothy Aebi, Maja Weisser, Evelyne Bucher, Hans H Hirsch, Stephan Marsch, Martin Siegemund

**Affiliations:** 1 Medical Intensive Care Unit, University Hospital Basel, Basel, Switzerland; 2 Department of Anesthesia and Surgical Intensive Care, University Hospital Basel, Basel, Switzerland; 3 Division of Infectious Diseases and Hospital Epidemiology, University Hospital Basel, Basel, Switzerland; 4 Institute for Medical Microbiology, Department of Biomedicine, University of Basel, Basel, Switzerland

## Abstract

**Background:**

Since the Influenza A pandemic in 1819, the association between the influenza virus and *Streptococcus pneumoniae *has been well described in literature. While a leading role has been so far attributed solely to Influenza A as the primary infective pathogen, Influenza B is generally considered to be less pathogenic with little impact on morbidity and mortality of otherwise healthy adults. This report documents the severe synergistic pathogenesis of Influenza B infection and bacterial pneumonia in previously healthy persons not belonging to a special risk population and outlines therapeutic options in this clinical setting.

**Case Presentation:**

During the seasonal influenza epidemic 2007/2008, three previously healthy women presented to our hospital with influenza-like symptoms and rapid clinical deterioration. Subsequent septic shock due to severe bilateral pneumonia necessitated intensive resuscitative measures including the use of an interventional lung assist device. Microbiological analysis identified severe dual infections of Influenza B with *Streptococcus pyogenes *in two cases and *Streptococcus pneumoniae *in one case. The patients presented with no evidence of underlying disease or other known risk factors for dual infection such as age (< one year, > 65 years), pregnancy or comorbidity.

**Conclusions:**

Influenza B infection can pose a risk for severe secondary infection in previously healthy persons. As patients admitted to hospital due to severe pneumonia are rarely tested for Influenza B, the incidence of admission due to this virus might be greatly underestimated, therefore, a more aggressive search for influenza virus and empirical treatment might be warranted. While the use of an interventional lung assist device offers a potential treatment strategy for refractory respiratory acidosis in addition to protective lung ventilation, the combined empiric use of a neuraminidase-inhibitor and antibiotics in septic patients with pulmonary manifestations during an epidemic season should be considered.

## Background

As early as 1903, French physician R. T. H. Laennec noted that the prevalence of pneumonia increased following an influenza epidemic [1]. This association between the influenza virus and *Streptococcus pneumoniae *became most obvious during the Influenza A pandemic of 1918, during which an estimated 40 to 50 million, mostly young and otherwise healthy people died, probably due to secondary bacterial pneumonia. Several reviews in the years after the pandemic led to the conclusion that bacteria were secondary pathogens and not the primary affecting agents [2]. There is evidence that the majority of deaths in the 1919 and subsequent 1957 and 1968 pandemics resulted directly from secondary bacterial pneumonia caused by common upper respiratory tract bacteria. These findings indicate, that addressing the viral cause alone by antiviral therapy and vaccination might not be sufficient, leading to a serious debate on pre-emptive empirical antibiotic therapy to cover the possibility of life-threatening secondary bacterial infections [3,4]. In regards of pandemic influenza planning, the importance of treating bacterial complications might be significantly higher in developing countries as neuraminidase inhibitors and vaccines might not be available to the majority of people [5].

So far a leading role has been attributed solely to Influenza A as the primary infective pathogen, while Influenza B is generally considered to be less pathogenic, having little impact on morbidity and mortality of otherwise healthy adults. We report for the first time severe secondary bacterial pneumonia with septic shock following infection with Influenza B in previously healthy women presenting to our hospital during the influenza season 2007/2008.

## Case Presentation

### Patient 1

A 39-year-old, previously healthy woman presented to the emergency unit with a 3-day history of myalgia, chills, high fever and a sore throat as well as vomitus, diarrhea and headaches. She was febrile, tachypnoeic with a respiratory rate of 40 per minute and showed an oxygen saturation of 92% despite receiving 10 litres of oxygen via face mask. She was tachycardic and hypotensive, indicating a septic shock. A patchy exanthema was visible on her trunk, and rales were noted upon bilateral auscultation. Her laboratory findings revealed a leucopenia of 2.9 ×10^-9^/l, a CRP of 507 mg/l and a procalcitonin of 139 ng/ml (Table 1). A chest x-ray showed diffuse infiltrates bilaterally.

**Table 1:** Characteristics of three previously healthy patients with primary Influenza B infection, severe bacterial pneumonia and septic shock

Characteristic	Patient 1	Patient 2	Patient 3
Date of admission	February 2008	March 2008	March 2008
Days of hospitalization	56	18	26
Age (years)	40	27	61
Co-morbidities	None	None	Osteoporosis
Septic shock *			
Tachycardia (beats/min)	160	120	110
Tachypnoea (rate/min)	40	45	30
Hypotension	Yes	Yes	Yes
Fever (°C)	40	39	39
Leukocyte count (×10S9/l)	2.96	12.4	14.2
Microbiology			
Initially isolated bacteria	S. pyogenes^†^	S. pyogenes^†^	S. pneumoniae^‡^
Influenza B RT-PCR ^§^	Positive	Positive	Positive
Influenza A RT-PCR ^§^	Negative	Negative	Negative
Initial therapy	Piperacillin/Tazobactam	Amoxicillin/clavulanic acid plus Clarithromycin	Ceftriaxon plus Clarithromycin
Final therapy	Penicllin plus	Penicillin plus	Vancomycin plus
	Oseltamivir	Oseltamivir	Oseltamivir
			
Laboratory findings ^||^			
CRP (mg/l)	507	445	329
PCT (ng/ml)	203	113	3.71
			
Arterial blood gas ^||^			
pH	7.19	7.28	7.45
paO2 (kPa)	8.7	9.72	6.59
paCO2 (kPa)	8.9	4.84	4.95
BE	-4.6	-8.8	2.5
Lactate (mmol/l)	1.4	5.1	2.5
Death on day	-	18	-

* Defined as patients showing all of following abnormal findings: tachycardia, tachypnoe, fever, leukocytosis or -penia and hypotension. Data printed are from the time of admission.

^† ^Results from bronchoalveolar lavage and blood cultures.

^‡ ^Result from urine pneumococcal antigen test.

^§ ^Real-time quantitative PCR from tracheal aspirate detecting Influenza A and B in separate reactions confirmed in at least two separate exams.

^|| ^Values showing the greatest deviation from normal during the first two days after admission. All blood gas data were obtained while applying high flow oxygen through a non re-breathing mask.

Blood cultures and expectorate were taken, and antibiotic therapy was initiated with intravenous ceftriaxone 2 g/day. Due to further respiratory deterioration, the patient was intubated. Aggressive volume replacement and high dose vasopressors were needed to stabilize the hypotensive shock. Haemofiltration was started for acute renal failure. After a diagnostic bronchoscopy with bronchoalveolar lavage on the day of admission, the antibiotic therapy was empirically changed to piperacillin/tazobactam 4.5 g 3 times daily and clarythromycin 500 mg twice daily. Oseltamivir 75 mg twice daily was added for 5 days, as the history was suspicious for influenza disease with bacterial superinfection. Due to progressive, refractory hypercapnia and respiratory acidosis, an interventional lung assist device (iLA membrane ventilator, Nova Lung^®^, Talheim, Germany) was installed the same day.^3^

*Group A streptococci *grew in four (of four drawn) blood cultures as well as in the bronchoalveolar lavage fluid. Taking the clinical picture into account, a streptococcal toxic shock syndrome was diagnosed, and the antibiotic therapy was changed to high-dose penicillin (5 Mio U four times a day). Clindamycin 900 mg three times daily was added to prevent toxin production for 3 days. A 5 day immunoglobulin therapy (Redimmune^® ^0.4 g/kg/d) was started. The bronchoalveolar lavage was repeated 5 days after admission to look for viral pathogens and confirmed the presence of Influenza B by real-time polymerase chain reaction (RT-PCR4) with 40,565 copies/ml (Table 1).

Over the following days, a nosocomial, ventilator-associated pneumonia was diagnosed and the antibiotic therapy was empirically changed to imipenem 500 mg four times a day. Methicillin susceptible *Staphylococcus aureus *was detected by culture of respiratory samples. Following clinical improvement, the lung assist device was removed after 11 days and the antibiotic therapy was stopped after 14 days. A third episode of respiratory deterioration occurred 2 weeks after admission. No pathogen could be isolated from bronchoalveolar lavage. The recurrence of severe respiratory acidosis due to refractory hypercarbia required another lung assist device, and a dilatational tracheotomy was performed.

After 24 days of intensive care, the patient's condition had finally improved enough for the lung assist device to be removed; the patient was weaned successfully and decanulated on day 35. The patient was transferred to a rehabilitation facility with mild critical illness polyneuropathy. She showed no neurological deficits otherwise.

### Patient 2

A previously healthy 27-year-old woman presented with complaints of an influenza-like infection with sore throat, fever and dry coughs since three days. The night before admission her sore throat became worse, fever climbed to 39°C and she complained of headaches. On admission, she was clinically diagnosed as having influenza, received intravenous fluids, and her discharge was planned thereafter. Within 8 hours post admission, her general condition deteriorated rapidly, showing progressive hypotension and respiratory failure. She was alert but in a bad general condition, fulfilling all criteria of a septic shock. Her laboratory findings on admission showed a leukocyte count of 12 ×10^-9^/l, a CRP of 103 mg/l and a procalcitonin level of 34 ng/ml (Table 1). A chest x-ray showed infiltrations in the right lung consistent with pneumonia. After blood cultures were drawn, an antibiotic therapy with amoxicillin/clavulanic acid 2.2 g three times daily and clarithromycin 500 mg twice daily was initiated and crystalloids and high dose catecholamines had to be applied. Due to respiratory failure, the patient was intubated. On the next morning, a marked pleural effusion with 1000 ml of exudative fluid was drained. Despite all these measures, her respiratory situation became worse with refractory hypoxaemia and increasing ventilation pressures, compelling us to install an interventional lung assist device. After this, her situation stabilized markedly. To this point, no other organ failures had occurred.

*Streptococcus pyogenes *grew in all four blood cultures drawn at admission and from the bronchoalveolar lavage. RT-PCR of the bronchoalveolar lavage for Influenza B was positive with 3,568 copies/ml and suggested a primary influenza infection with bacterial super infection (Table 1). Antibiotic treatment was changed to high-dose penicillin (5 Mio U four times a day) and oseltamivir 75 mg twice daily was added (on day 5 after admission) for 5 days. On day seven, ventilator associated pneumonia was suspected and bronchoalveolar lavage repeated. No causative agent was identified, and another Influenza RT-PCR was negative for Influenza B.

Fifteen days after admission, a sudden respiratory deterioration occurred with pulmonary hemorrhage, compounding mechanical ventilation markedly. A bronchoscopy showed crustal lesions in the trachea but no active bleeding. On suspicion of pneumothoraces, thoracic drainages were inserted bilaterally. A malfunction of the interventional lung assist device made a change of the device necessary. The invasive procedure was poorly tolerated, and temporary mechanical resuscitation had to be performed. Thereafter, the patient remained dependant on high dose catecholamines and showed persistent signs of cerebral hypoxaemia. The patient died 18 days after admission. Autopsy was declined by the family for religious reasons.

### Patient 3

A 61-year-old previously healthy woman was admitted to hospital after returning from a one week of holiday in Morocco. She complained of headaches, a sore throat, no appetite and general weakness since 9 days, and mentioned having diarrhoea since 6 days. She was started on amoxicillin 750 mg three times daily by a family doctor without effect. The day she presented to our emergency unit she was febrile with 38°C and tachypnoeic. Auscultation detected rales bilaterally and bilateral infiltrates were found on a chest x-ray. Her laboratory findings showed a leukocyte count of 14 ×10^-9^/l and a CRP of 195 mg/l initially (Table 1). An empiric antibiotic therapy with ceftriaxone 2 g/day and clarythromycin 500 mg twice daily was initiated, and the septic patient was transferred to the intensive care unit. The pneumococcal antigen was positive in the urine sampled on admission. Blood cultures remained negative, which may be explained by the antibiotic pretreatment. A direct influenza antigen test was negative initially.

Due to respiratory failure, the patient was intubated on day 4 despite non-invasive ventilation. A spontaneous right-sided pneumothorax was treated with a thoracic drainage. Over the next few hours, her respiratory situation deteriorated further showing a progressive hypoxaemia and a reduced lung compliance requiring an interventional lung assist device. With the suspicion of penicillin-resistant *pneumococci *in this patient returning from Morocco, the antibiotic therapy was changed to vancomycin 1 g twice daily. A second urine test for pneumococcal antigen was again positive. No other bacteria was detected in a bronchial alveolar lavage five days after admission, but RT-PCR from a bronchial alveolar lavage on day 5 revealed Influenza B with 12,231 copies/ml (Table 1). Oseltamivir 75 mg twice daily was added (on day 9 after admission) for 5 days, and the RT-PCR repeated thereafter remained positive with 13,363 copies/ml for Influenza B. Seven days after admission, the pulmonary situation had stabilized enough that the interventional lung assist device could be safely removed. One day later she was extubated. The patient was transferred to a rehabilitation facility 26 days after admission.

This is the first report of Influenza B infections accompanied by severe bacterial superinfection in three healthy women.

Fatal co-infections of influenza virus with *Streptococcus pneumoniae *have been described in mice since the early eighties [6], and have been reported following in clinical outbreaks [7]. In addition to *S. pneumoniae*, the most common co-infecting bacteria are *Staphylococcus aureus*, *Haemophilus influenzae*, *Streptococcus pyogenes *and *Mycoplasma pneumoniae*. Pathophysiologically various data from mice models suggests that influenza virus infection damages the epithelium of the bronchi and lungs, allowing microaspirated bacterial pathogens to establish infection [8]. On the other hand, influenza alters the local immunity[9] and the inflammatory response [10-12] facilitating the outgrowth of bacteria. Underlying immunosuppression might lead to more severe and prolonged disease [13]. Our patients presented with no evidence of underlying disease or other known risk factors for dual infection such as age (< one year, > 65 years), pregnancy or comorbidity [14].

**Figure 1:**
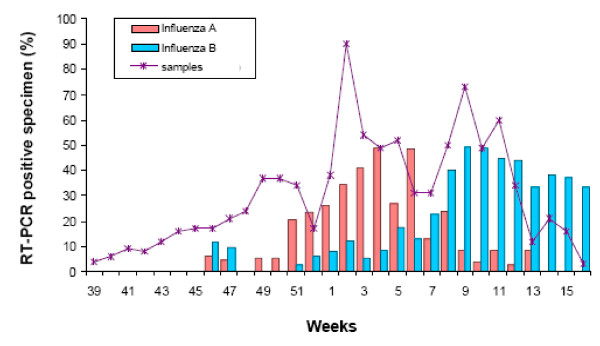
**Distribution of influenza subtypes recorded by the influenza surveillance sentinel network in Switzerland (Season 2007 - 2008)^15^**.

**Figure 2:**
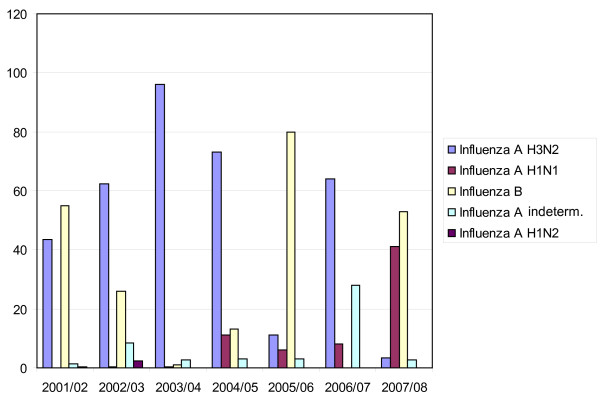
**Percentage of the various influenza subtypes recorded by the influenza surveillance sentinel network in Switzerland (Season 2001 - 2008)^15^**.

From an epidemiological viewpoint, the circulating strain is known to contribute to mortality with H3N2 subtype causing more influenza-associated deaths than H1N1 or influenza B viruses [15]. Influenza B leads to fewer co-infections [16] and milder disease compared to Influenza A [8,17]. In Switzerland, the seasonal influenza epidemics of 2007/2008 were characterized by an initial increase in Influenza A H3N2 cases, whereas in the later months, when our patients presented, infections with Influenza B prevailed [17] (Figure 1). The predominant circulating B strain in Switzerland was B/Jiangsu/10/03. This strain was not matched by a corresponding strain in the B component of the vaccine and was not present in the previous year (Figure 2), therefore, it is possible that there is no immunologic memory for this infection, perhaps contributing to the severity of infection [18]. The first and second patient had young children suffering from influenza-like symptoms and most probably transmitted the virus to their mother. In general, we do not think that there is an association between sex and disease severity. While pregnancy was associated with poor outcome in the H1N1 pandemics, none of our patients was pregnant.

Diagnosis in our cases was based on real-time quantitative PCR, according to a previously published protocol detecting Influenza A and B in separate reactions with a limit of detection of 2.5 log10 copies/ml [19]. This method is known to be very sensitive and specific compared to antigen testing, which is a more rapid but less sensitive test, and was negative in one of our three patients. Severe cases of Influenza B have probably always occurred. But as most routine laboratories did no specific testing for Influenza B, this could not be documented until recently. Interestingly, a recent study on the effect of oseltamivir in patients hospitalized with Influenza infection included 215 patients with influenza B, of which 13 died, confirming our data on serious courses of Influenza B infections [20].

All three patients received oseltamivir, a highly selective neuraminidase-inhibitor. This agent is effective against Influenza A and B. Although clinical data are limited in Influenza B, some evidence exists that oseltamivir might have less effect on Influenza B, therefore, higher dosing may be necessary [21]. Patient 1 received an early empiric treatment with oseltamivir, as her history was indicative for primary influenza infection and bacterial superinfection. In the other two patients, antiviral treatment was started only later, on day 5 in the second and at day 9 in the third patient. Given the fact that antiviral treatment is efficacious especially in the early course, empirical use of oseltamivir in severe pneumonia during an influenza season might be advisable, even though PCR-testing is a highly sensitive and reasonably rapid. With regard to oseltamivir's properties and the fact that septic patients are immunocompromised per se, the early use of neuraminidase-inhibitors seems warranted. In fact, our case series favours the approach of empirical combined antiviral-antibiotic treatment, a discussion that evolved during the H1N1 pandemics. However, there is no evidence that the use of oseltamivir yields any benefit after 48 hours of infection.

Supportive care measures for septic shock and respiratory failure were taken immediately. Minimal inhibitory concentrations for penicillin were low in the two cases with *S. pyogenes *indicating full susceptibility towards the administered empirical betalactam antibiotic. To prevent toxin production, clindamycin was added. *S. pyogenes *is known to cause fulminant toxic shock syndromes, for which both patients qualify, occurring also in young and previously healthy persons [22]. In the third case, no resistance testing was available due to diagnosis of *S. pneumoniae *by means of a non-cultural assay only. A penicillin-resistant strain might have been possible in the patient returning from Morocco, where the local epidemiology shows up to 40% penicillin-resistant pneumococci [23] and, therefore, may have contributed to delay in a correct antibiotic treatment.

The application of an interventional lung assist enables a safe application of lung protective ventilation [24] together with a therapy of respiratory acidosis in patients where all other intensive care measures have failed to provide adequate ventilation and decarboxylation. Although uncommonly used on our ward, the lung assist device allowed us to combine very low tidal volumes (3-6 ml/kg) and high PEEP levels with lower plateau pressures and, thus, the avoided barotraumas reported in these patients. Especially during acute lung injury due to pneumonia, the application of low tidal ventilation may prevent secondary injury from mechanical ventilation to the uninfected parts of the lung.

## Conclusions

Influenza B infection can pose a risk for severe secondary infection in previously healthy persons. As patients admitted to hospital due to severe pneumonia are rarely tested for Influenza B, the incidence of admission due to this virus might be greatly underestimated, therefore, a more aggressive search for influenza virus and empirical treatment might be warranted. While the use of an interventional lung assist device offers a potential treatment strategy for refractory respiratory acidosis in addition to protective lung ventilation, the combined empiric use of a neuraminidase-inhibitor and antibiotics in septic patients with pulmonary manifestations during an epidemic season should be considered.

## Competing interests

The authors declare that they have no competing interests.

## Authors' contributions

TA, EB, SM and MS identified the patients, reviewed their patient charts and performed a literature research. MW and HHH worked off the infectiological background and provided figure 1 and figure 2. MS provided additional information on interventional lung assist devices. TA drafted the manuscript. All authors read and approved the final version of the manuscript.

## Pre-publication history

The pre-publication history for this paper can be accessed here:

http://www.biomedcentral.com/1471-2334/10/308/prepub
